# Complete genome sequence of bacteriochlorophyll-synthesizing bacterium *Porphyrobacter neustonensis* DSM 9434

**DOI:** 10.1186/s40793-017-0243-5

**Published:** 2017-05-10

**Authors:** Qian Liu, Yue-Hong Wu, Hong Cheng, Lin Xu, Chun-Sheng Wang, Xue-Wei Xu

**Affiliations:** 1 Key Laboratory of Marine Ecosystem and Biogeochemistry, Second Institute of Oceanography, State Oceanic Administration, 36th Baochubei Road, Hangzhou, 310012 People’s Republic of China; 2 College of Life Sciences, Zhejiang University, Hangzhou, 310058 People’s Republic of China

**Keywords:** *Porphyrobacter neustonensis* DSM 9434, Aerobic anoxygenic phototrophic bacteria, Bacteriochlorophyll synthesis, Genome sequence, *Alphaproteobacteria*

## Abstract

The genus *Porphyrobacter* belongs to aerobic anoxygenic phototrophic bacteria cluster. *Porphyrobacter neustonensis* DSM 9434 was isolated from a eutrophic freshwater pond in Australia, and is able to synthesize Bacteriochlorophyll *a* as well as grow under aerobic conditions. It is the type species of the genus *Porphyrobacter*. Here we describe the characteristics of the strain DSM 9434, including the genome sequence and annotation, synthesis of BChl *a*, and metabolic pathways of the organism. The genome of strain DSM 9434 comprises 3,090,363 bp and contains 2,902 protein-coding genes, 47 tRNA genes and 6 rRNA genes. Strain DSM 9434 encodes 46 genes which participate in BChl *a* synthesis and this investigation shed light on the evolution and functional implications regarding bacteriochlorophyll synthesis.

**Electronic supplementary material:**

The online version of this article (doi:10.1186/s40793-017-0243-5) contains supplementary material, which is available to authorized users.

## Introduction

Aerobic anoxygenic phototrophic bacteria probably evolved after the accumulation of oxygen in the earth’s biosphere [1]. They are widely distributed in the euphotic zone of the ocean as well as terrestrial water, and play an ecologically and biogeochemical important role in aquatic systems, especially marine carbon cycling [2–4]. AAP bacteria harvest light by Bacteriochlorophyll *a* and possess various carotenoids as auxiliary pigments [5]. They derive a significant portion of their energy requirements from light but perform photoheterotrophic metabolism based on an obligatory supply of organic substrates for growth [6]. Until now, all the AAP bacteria that have been discovered belong to the *Proteobacteria*, and the majority of cultured AAP strains are members of the *Alphaproteobacteria* [5].

*Porphyrobacter* has been proposed as a genus along with four *Porphyrobacter* strains being isolated from a eutrophic freshwater pond in Australia [7]. They are obligate aerobes in the AAP bacteria cluster. *Porphyrobacter neustonensis* strain DSM 9434 is the type strain of the genus *Porphyrobacter* [7]. To get insight into the capability of *Porphyrobacter* in adapt to harvest energy photosynthetically, recently, we obtained the complete genome of *P. neustonensis* strain DSM 9434 and detected key genes for synthesizing BChl *a* and mediating aerobic anoxygenic phototrophic metabolism. We also describe the genomic sequencing related to its annotation for understanding their physiological, metabolic and ecological functions in the environments.

## Organism information

### Classification and features

**Fig. 1:**
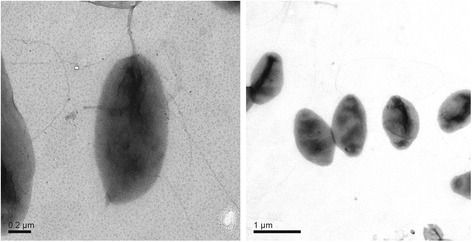
Transmission electron microscopy of cells of *Porphyrobacter neustonensis* DSM 9434. The peritrichous flagella are present. Bars represent scales of 0.2 μm (**a**) and 1 μm (**b**), respectively

**Fig. 2:**
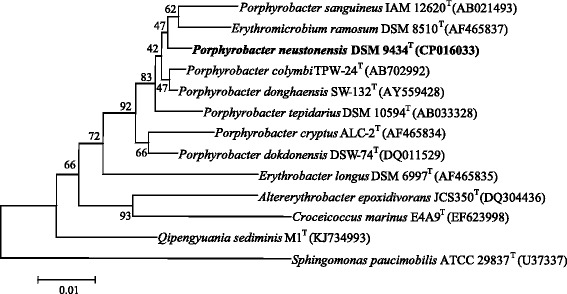
Phylogenetic tree based on 16S rRNA gene sequences was constructed by neighbor-joining algorithms. Related sequences were aligned with Clustal W [21]. Evolutionary distances were calculated according to the algorithm of the Kimura two-parameter model with bootstraps analysis set to 1000 replicates. Bar, 0.01 substitutions per nucleotide position

*P. neustonensis* DSM 9434 was purified from a peptone-yeast extract alga plate after being isolated from the euphotic freshwater pond in Australia [7]. The strain grew with temperature between 10 and 37 °C [7]. The cell is rod-shaped, and occasionally coccoid and ovoid (Fig. 1). The strain produced BChl *a* and carotenoid*,* analyzed by extracting cells with ethanol (Additional file 1: Figure S1). It grew aerobically in the dark and used a series of organic carbon, such as galactose, glucose, maltose, mannose, sucrose, xylose, arginine, as sole sources of carbon and energy [7]. Analysis of cell wall materials isolated from strain DSM 9434 detected muramic acid and diaminopimelic acid, the major components of peptidoglycan cell wall layer [7]. A high proportion of fatty acids identified as octadecenoic acids (18:1, 84%) is present in the cell with minor components of fatty acids, such as octadecadienoic acid (18:2, 6.1%), 2-hydroxytetradecanoic acid (2OH14:0, 2.7%) and hexadecanoic acid (16:0, 2.6%) [7]. Based on phylogenetic analysis of 16S rRNA gene sequence, the strain belongs to the *Alphaproteobacteria* class and falls into the cluster comprising the *Porphyrobacter* species (Fig. 2). The classification and features of *P. neustonensis* DSM 9434 are summarized in Table 1.

**Table 1:** Classification and general features of *Porphyrobacter neustonensis* DSM 9434 according to the MIGS recommendations [22]

MIGS ID	Property	Term	Evidence code^a^
	Classification	Domain *Bacteria*	TAS [23]
		Phylum *Proteobacteria*	TAS [24]
		Class *Alphaproteobacteria*	TAS [25, 26]
		Order *Sphingomonadales*	TAS [25, 27]
		Family *Erythrobacteraceae*	TAS [28]
		Genus *Porphyrobacter*	TAS [7]
		Species *Porphyrobacter neustonensis*	TAS [7]
		Type strain *DSM 9434*	
	Gram stain	Negative	IDA
	Cell shape	Rod or cocci	IDA
	Motility	Motile	IDA
	Sporulation	Non-sporulation	IDA
	Temperature range	10–37 °C	TAS [7]
	Optimum temperature	Not reported	
	pH range; Optimum	Not reported	
	Carbon source	Organic carbon	TAS [7]
MIGS-6	Habitat	Freshwater	TAS [7]
MIGS-6.3	Salinity	Not reported	
MIGS-22	Oxygen requirement	Strictly aerobic	TAS [7]
MIGS-15	Biotic relationship	free-living	TAS [7]
MIGS-14	Pathogenicity	Non-pathogen	NAS
MIGS-4	Geographic location	University of Queensland, Australia	TAS [7]
MIGS-5	Sample collection	Not reported	
MIGS-4.1	Latitude	Not reported	
MIGS-4.2	Longitude	Not reported	
MIGS-4.4	Altitude	Sea level	NAS

^a^Evidence codes - *IDA* Inferred from Direct Assay, *TAS* Traceable Author Statement (i.e., a direct report exists in the literature), *NAS* Non-traceable Author Statement (i.e., not directly observed for the living, isolated sample, but based on a generally accepted property for the species, or anecdotal evidence). These evidence codes are from the Gene Ontology project

## Genome sequencing information

### Genome project history

*P. neustonensis* DSM 9434 was selected for sequencing in the project of *Porphyrobacter* Genome Sequencing and Assembly because it is relevant to genomic sequencing of the whole family of *Erythrobacteraceae* and BChl *a* synthesis. The complete genome sequence was finished on May 31, 2016 and presented for public access on June 22, 2016. This whole genome has been deposited at DDBJ/EMBL/GenBank under the accession number CP016033. The main genome sequence information is present in Table 2.

**Table 2:** Genome sequencing project information

MIGS ID	Property	Term
MIGS 31	Finishing quality	Finished
MIGS-28	Libraries used	10 kb
MIGS 29	Sequencing platforms	A PacBio RS II platform
MIGS 31.2	Fold coverage	203-fold
MIGS 30	Assemblers	HGAP Assembly version 2, Pacific Biosciences
MIGS 32	Gene calling method	RAST
	Locus Tag	A9D12
	Genbank ID	CP016033
	GenBank Date of Release	June 22, 2016
	GOLD ID	Go0029942
	BIOPROJECT	PRJNA322640
MIGS 13	Source Material Identifier	DSM (Deutsche Sammlung von Mikroorganismen und Zellkulturen GmbH)
	Project relevance	Bacteriochlorophyll *a* synthesis

### Growth conditions and genomic DNA preparation

*P. neustonensis* DSM 9434 was aerobically cultivated in Luria-Bertani medium at 28 °C. High-quality genomic DNA was extracted using Qiagen DNA extraction kit based on its protocol. DNA sequencing of *P. neustonensis* DSM 9434 was performed using SMRT technology. One Library with insert size of 10 kb was constructed according to the large SMRTbell gDNA protocol (Pacific Biosciences, USA).

### Genome sequencing and assembly

Genomic DNA was sequenced with a PacBio RS II platform yielding 48,527 reads with an average length of 12,972 nt (600 Mb, 203-fold genome coverage; Pacific Biosciences). These reads were assembled using HGAP Assembly version 2 (Pacific Biosciences, USA). The final contigs were checked for circularization and the overlapping ends were trimmed.

### Genome annotation

The tRNA genes were identified using tRNAscan-SE 1.21 [8] with bacterial model, and rRNA genes were found via RNAmmer 1.2 Server [9]. The open reading frames (ORFs) and the functional annotation of translated ORFs were predicted and achieved by using the RAST server online [10]. Classification of some predicted genes and pathways were analyzed using COG database [11] and KEGG database [12, 13].

## Genome properties

**Fig. 3:**
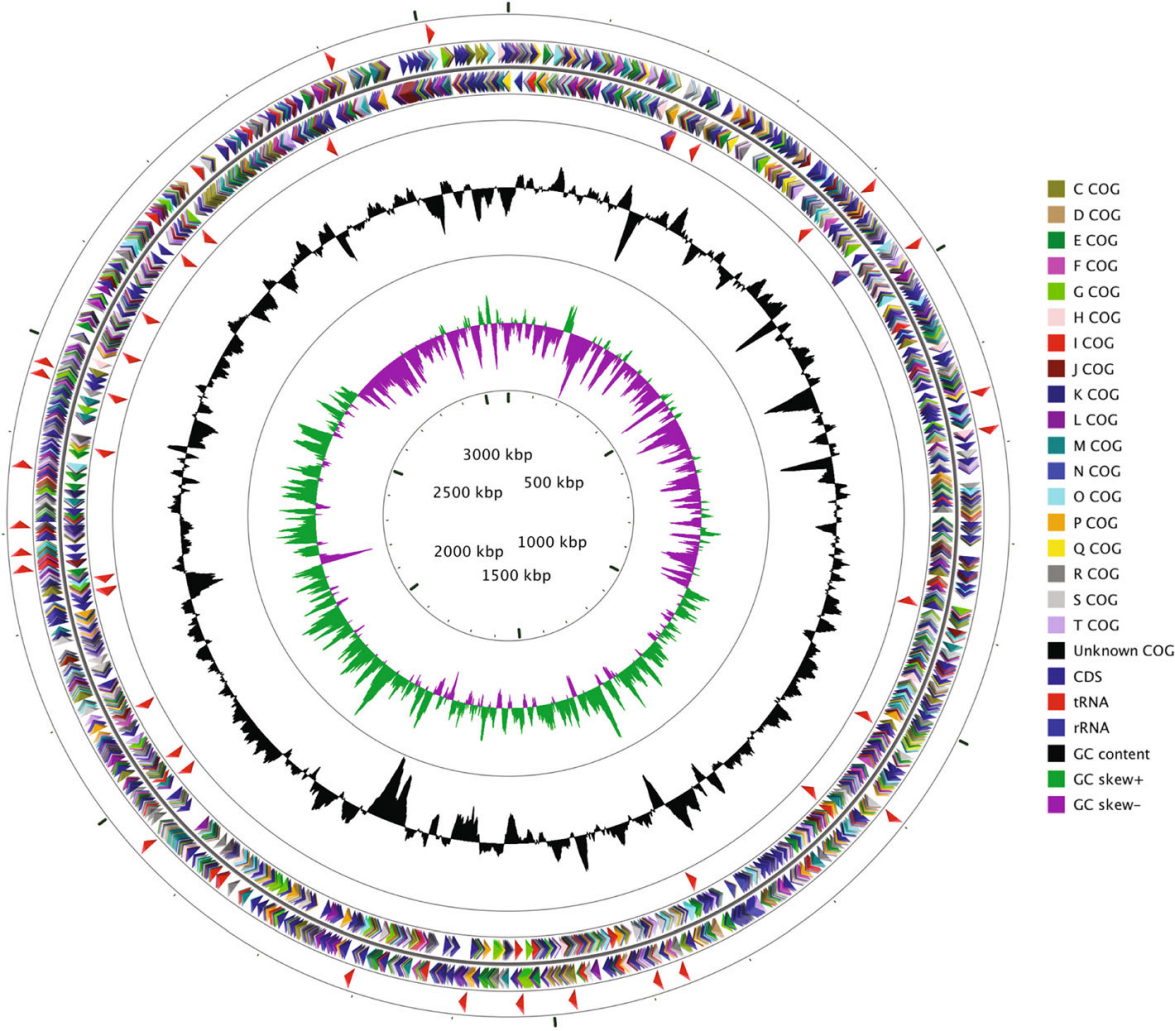
Circular map of the chromosome of *Porphyrobacter neustonensis* DSM 9434. From outside to the center: RNA genes on the forward strand (tRNAs *red*, rRNAs *blue*), genes on the forward strand (colored by COG categories), genes on the reverse strand (colored by COG categories), RNA genes on the reverse strand (tRNAs *red*, rRNAs *blue*), G + C content (peaks out/inside the circle indicate values higher or lower than the average G + C content, respectively), GC skew (calculated as (G-C)/(G + C), *green/purple* peaks out/inside the circle indicate values higher or lower than 1, respectively), genome size (3,090,363 bp)

The genome of strain DSM 9434 contains a single circular chromosome (Fig. 3). The complete genome of strain DSM 9434 comprises 3,090,363 bp with an average G + C content of 65.3%. The contig contains 2,902 coding sequences of total 2955 genes, 47 tRNAs and 2 operons of 16S-23S-5S rRNA gene. The summary of features and statistics of the genome is shown in Table 3 and genes belonging to COG functional categories are listed in Table 4.

**Table 3:** Genome statistics

Attribute	Value	% of Total
Genome size (bp)	3,090,363	100
DNA coding (bp)	2,809,376	90.91
DNA G + C (bp)	2,016,518	65.25
DNA scaffolds	1	-
Total genes	2955	100
Protein coding genes	2902	98.21
RNA genes	53	1.79
Pseudo genes	-	-
Genes in internal clusters	350	11.84
Genes with function prediction	2189	74.08
Genes assigned to COGs	2326	78.71
Genes with Pfam domains	2373	80.30
Genes with signal peptides	400	13.54
Genes with transmembrane helices	674	22.81
CRISPR repeats	2	-

**Table 4:** Number of genes associated with general COG functional categories

Code	Value	% age	Description
J	158	6.13	Translation, ribosomal structure and biogenesis
A	2	0.08	RNA processing and modification
K	129	5.00	Transcription
L	110	4.27	Replication, recombination and repair
B	4	0.16	Chromatin structure and dynamics
D	25	0.97	Cell cycle control, Cell division, chromosome partitioning
V	43	1.67	Defense mechanisms
T	165	6.40	Signal transduction mechanisms
M	167	6.48	Cell wall/membrane biogenesis
N	61	2.37	Cell motility
U	87	3.37	Intracellular trafficking and secretion
O	117	4.54	Posttranslational modification, protein turnover, chaperones
C	169	6.56	Energy production and conversion
G	97	3.76	Carbohydrate transport and metabolism
E	172	6.67	Amino acid transport and metabolism
F	63	2.44	Nucleotide transport and metabolism
H	123	4.77	Coenzyme transport and metabolism
I	152	5.90	Lipid transport and metabolism
P	127	4.93	Inorganic ion transport and metabolism
Q	79	3.06	Secondary metabolites biosynthesis, transport and catabolism
R	286	11.09	General function prediction only
S	242	9.39	Function unknown
-	546	19.01	Not in COGs

## Insights from the genome sequence

### Bacteriochlorophyll *a* synthesis and phototropic activity

The genome of *P. neustonensis* DSM 9434 harbors 46 genes which participate in BChl *a* synthesis (Additional file 2: Table S1). A complete photosynthesis gene cluster structures was observed. The PGC is 38 kb and includes 5 main sets of genes: *bch* genes encoding enzymes involved in the BChl *a* biosynthetic pathway, *puf* operons encoding proteins forming the reaction center, *puh* operons involved in the RC assembly, *crt* genes responsible for biosynthesis of carotenoids and a variety of regulatory genes. The complete PGC in the genome of *P. neustonensis* DSM 9434 genome consists of *bch*IDO-*crt*CDF-bchCXYZ-*puf*ALM-*tsp*O-*bch*P-*bch*G-*pps*R-*ppa*A-*bch*FNBHLM-*lha*A*-puh*ABC-*asc*F-*puh*E-*hem*A-*cyc*A (Additional file 2: Table S1).

The heart of aerobic anoxygenic phototrophy is the RC encoded by the *puf* and *puh* operons. The *puf* operon encodes the subunits of the light-harvesting (LH1) (*puf*A, ANK11803) and RC complex (*puf*L and *puf*M, ANK11804 -11805). The *puh* operons encoding RC assembly indirectly effect on LH1 assembly (*puh*ABC, ANK11818-11820, *puh*E, ANK11823). Gene *lha*A (ANK11817) encodes a possible LH1 assembly protein [14]. Genes *bch*BCDFGHILMNOPXYZ (ANK11793-11795, 11800–11802, 13992, 11806, 11808, 11811–11816) and *asc*F (ANK11822), with exception of 8-vinyl reductase (ANK12775), represent the complete biosynthetic pathway from protoporphyrin XI to BChl *a*. The cluster of three carotenoid biosynthesis genes, *crtC* (ANK11797), *crtD* (ANK11798) and *crtF* (ANK11799) may participate in the formation of acyclic xanthophylls from lycopene [15]. Other carotenoid biosynthesis genes are located outside the cluster (*crtE*, ANK13491; *crtB*, ANK12836; *crtI*, ANK14187; *crtY*, ANK14188; *crtZ*, ANK11768; *crtW*, ANK13982, 14112 and 13340). Three regulatory genes (*ppsR*, *ppaA* and *tspO*) were found in the genome of strain DSM 9434. Regulatory genes *ppsR* (DNA-binding repressor, ANK11809) and *ppa*A (oxygen sensor, ANK11810) are sensitive to light intensity and oxygen concentration [16], and the gene *tsp*O (tryptophan-rich sensory protein precursor, ANK13994) negatively affects the transcriptional expression of several photosynthesis genes [17].

### Metabolism of *P. neustonensis*DSM 9434

The complete genome of *P. neustonensis* DSM 9434 was annotated for understanding the major metabolic pathways of carbon, nitrogen, sulfur and phosphorus based on the key genes it processes. As we mentioned, although it has bacteriochlorophyll-synthesis genes and acquires energy from light, the absence of carbon fixation and CO-oxidizing genes indicates that strain DSM 9434 is not able to grow autotrophically. They can only use organic carbon sources. It does not have a complete glycolysis pathway but processes key genes for the Entener-Doudoroff, the pentose phosphate pathway, and the tricarboxylic acid cycle. The genome of *P. neustonensis* DSM 9434 harbors a variety of transporter genes for ammonium (*amt*B) and other organic nitrogen substrates (e.g. amino acids, polyamines). It is lack of genes involved in nitrate/nitrite reduction, nitrogen fixation or anaerobic ammonium oxidation, thus strain DSM 9434 only relies on reduced nitrogen sources. The genes encoding urea transporter and urease *(ure*ABC*)* are absent in the genome of DSM 9434, suggesting its incapability of utilizing urea as a C or N source in the environment. The lack of urea uptake and degradation may reflect the environmental adaption of strain DSM 9434 from a eutrophic pond, where ammonium and algae-derived organic N (e.g. amino acids and polyamines) are usually enriched [18, 19]. *P. neustonensis* DSM 9434 processes genes involved in assimilatory SO_4_ reduction (e.g. *sul*P encoding sulfate permease). Sulfate can be reduced to sulfide (*cys*), subsequently being incorporated into amino acids. The strain DSM 9434 is also able to utilize organic sulfur compounds (e.g. amino acids, alkanesulfonates); however, it missed the transporter genes (*ssu*ACB) for uptake of extracellular alkanesulfonates. Strain DSM 9434 possesses the high-affinity phosphate transporter (*pst*SCAB) and regulatory genes (*pho*UBR), and genes for inorganic P storage as polyphosphate (*ppk*), a signal of using an alternative strategy for maintaining a phosphate supply [20]. The presence of genes encoding alkaline phosphatase in the genome of strain DSM 9434 indicates that it is capable of using both inorganic and organic forms of phosphorus.

## Conclusion

The complete genome sequence of the BChl *a* synthesizing bacteria *P. neustonensis* DSM 9434 provide an insight into the genomic basis of its metabolic characteristics and bacteriochlorophyll-synthesis pathway. This investigation sheds light on the evolution of PGCs of aerobic anoxygenic phototrophs and provides the possibility for comparative genomics of AAP bacteria isolated from marine, freshwater and terrestrial environments.

## Additional files

Additional file 1: Figure S1. *Porphyrobacter neustonensis* DSM 9434. In vitro absorption spectrum of ethanol extract of showing peaks of carotenoid (452–484 nm) and Bacteriochlorophyll *a* (765 nm). (EPS 1267 kb)

Additional file 2: Table S1. Genes related to bacteriochlorophyll-synthesis in the genome of *Porphyrobacter neustonensis* DSM 9434. (DOCX 35 kb)

## Abbreviations

AAP: Aerobic anoxygenic phototrophic
BChl *a*: Bacteriochlorophyll *a*
COG: Clusters of Orthologous Groups
LH1: Light-harvesting
ORF: Open reading frame
PGC: Photosynthesis gene cluster
RC: Reaction center
