# Supplementary material for: Complete genome sequence of bacteriochlorophyll-synthesizing bacterium Porphyrobacter neustonensis DSM 9434
Source: Stand Genomic Sci. 2017 May 10;12:32. doi: 10.1186/s40793-017-0243-5 (PMC5424368; doi:10.1186/s40793-017-0243-5)
Supplement: Supplementary file 2 — Genes related to bacteriochlorophyll-synthesis in the genome of Porphyrobacter neustonensis DSM 9434. (DOCX 35 kb) [file 40793_2017_243_MOESM2_ESM.docx]

Additional file 2: Table S1. Genes related to bacteriochlorophyll-synthesis in the genome of *Porphyrobacter neustonensis* DSM 9434

| Enzyme gene | Protein Id | Start | Stop | Strand | Size/aa | Predicted function* |
| --- | --- | --- | --- | --- | --- | --- |
| / | ANK12775 | 1553810 | 1554694 | + | 294 | Divinyl protochlorophyllide a 8-vinyl-reductase (EC 1.3.1.75) |
| *bch*I | ANK11793 | 256791 | 257792 | + | 333 | Protoporphyrin IX Mg-chelatase subunit I (EC 6.6.1.1) |
| *bch*D | ANK11794 | 257789 | 259474 | + | 561 | Protoporphyrin IX Mg-chelatase subunit D (EC 6.6.1.1) |
| *bch*O | ANK11795 | 259471 | 260361 | + | 296 | Predicted hydrolases or acyltransferases (alpha/beta hydrolase superfamily) |
| Hyp | ANK11796 | 260569 | 261618 | + | 349 | Hypothetical protein |
| Hyp | ANK13991 | 261623 | 262696 | + | 357 | Hypothetical protein |
| *crt*C | ANK11797 | 263736 | 262984 | - | 250 | Hydroxyneurosporene synthase |
| *crt*D | ANK11798 | 265388 | 263823 | - | 521 | Methoxyneurosporene dehydrogenase (EC 1.14.99.-) |
| *crt*F | ANK11799 | 265491 | 266615 | + | 374 | Hydroxyneurosporene methyltransferase (EC 2.1.1.-) |
| *bch*C | ANK11800 | 266743 | 267690 | + | 315 | 2-Desacetyl-2-hydroxyethyl bacteriochlorophyllide A dehydrogenase BchC |
| *bch*X | ANK13992 | 267690 | 268679 | + | 329 | Chlorophyllide reductase subunit BchX (EC 1.18.-.-) |
| *bch*Y | ANK11801 | 268676 | 270208 | + | 510 | Chlorophyllide reductase subunit BchY (EC 1.18.-.-) |
| *bch*Z | ANK11802 | 270205 | 271650 | + | 481 | Chlorophyllide reductase subunit BchZ (EC 1.18.-.-) |
| *puf*A | ANK11803 | 272062 | 272250 | + | 62 | Light-harvesting LHI, alpha subunit |
| *puf*L | ANK11804 | 272351 | 273175 | + | 274 | Photosynthetic reaction center L subunit |
| *puf*M | ANK11805 | 273190 | 274161 | + | 323 | Photosynthetic reaction center M subunit |
| Hyp | ANK13993 | 274551 | 274297 | - | 84 | Hypothetical protein |
| *tsp*O | ANK13994 | 275068 | 274601 | - | 155 | Tryptophan-rich sensory protein precursor |
| *bch*P | ANK11806 | 276332 | 275139 | - | 397 | Geranylgeranyl hydrogenase BchP; Geranylgeranyl diphosphate reductase (EC 1.3.1.83) |
| ORF | ANK11807 | 277687 | 276329 | - | 452 | Bacteriochlorophyll synthase 44.5 kDa chain |
| *bch*G | ANK11808 | 278619 | 277687 | - | 310 | Chlorophyll a synthase ChlG (EC 2.5.1.62) |
| *pps*R | ANK11809 | 280345 | 278924 | - | 473 | Regulator of carotenoid biosynthesis; Transcriptional regulator, PpsR |
| *ppa*A | ANK11810 | 281258 | 280356 | - | 300 | Heme-binding SCHIC domain protein, putative oxygen sensor |
| *bch*F | ANK11811 | 281547 | 282050 | + | 167 | 2-Vinyl bacteriochlorophyllide hydratase BchF (EC 4.2.1.-) |
| *bch*N | ANK11812 | 282047 | 283351 | + | 434 | Light-independent protochlorophyllide reductase subunit N (EC 1.18.-.-) |
| *bch*B | ANK11813 | 283355 | 284932 | + | 525 | Light-independent protochlorophyllide reductase subunit B (EC 1.18.-.-) |
| *bch*H | ANK11814 | 284922 | 288563 | + | 1213 | Protoporphyrin IX Mg-chelatase subunit H (EC 6.6.1.1) |
| *bch*L | ANK11815 | 288582 | 289478 | + | 298 | Light-independent protochlorophyllide reductase iron-sulfur ATP-binding protein ChlL (EC 1.18.-.-) |
| *bch*M | ANK11816 | 289478 | 290182 | + | 234 | Mg-Protoporphyrin O-methyltransferase (EC 2.1.1.11) |
| *lha*A | ANK11817 | 290179 | 291624 | + | 481 | PucC protein |
| *puh*A | ANK11818 | 291694 | 292500 | + | 268 | Photosynthetic reaction center H subunit |
| *puh*B | ANK11819 | 292491 | 293267 | + | 258 | Putative photosynthetic complex assembly protein |
| *puh*C | ANK11820 | 293264 | 293752 | + | 162 | InterPro IPR001092 |
| Hyp | ANK11821 | 293752 | 294054 | + | 100 | Hypothetical protein |
| *acs*F | ANK11822 | 294051 | 295112 | + | 353 | Mg-Protoporphyrin IX monomethyl ester oxidative cyclase (aerobic) (EC 1.14.13.81) |
| *puh*E | ANK11823 | 295130 | 295903 | + | 257 | Hypothetical protein |
| *hem*A | ANK11824 | 295933 | 297153 | + | 406 | 5-Aminolevulinate synthase (EC 2.3.1.37) |
| *cyc*A | ANK13995 | 298509 | 297367 | - | 380 | Beta-lactamase (EC 3.5.2.6) |
| *crtE* | ANK13491 | 2532092 | 2533003 | + | 303 | Geranylgeranyl pyrophosphate synthetase (EC 2.5.1.29) |
| *crtB* | ANK12836 | 1649636 | 1648671 | - | 321 | Phytoene synthase (EC 2.5.1.32) |
| *crtI* | ANK14187 | 1652113 | 1650662 | - | 483 | Phytoene dehydrogenase (EC 1.14.99.-) |
| *crtY* | ANK14188 | 1653498 | 1652221 | - | 425 | Lycopene cyclase |
| *crtZ* | ANK11768 | 221937 | 221398 | - | 179 | Beta-carotene hydroxylase |
| *crtW* | ANK13982 | 222737 | 221934 | - | 267 | Beta-carotene ketolase (EC 1.14.-.-) |
| *crtW* | ANK14112 | 1113816 | 1115423 | + | 535 | Beta-carotene ketolase (EC 1.14.-.-) |
| *crtW* | ANK13340 | 2313577 | 2312522 | - | 351 | Beta-carotene ketolase (EC 1.14.-.-) |

*Based on RAST results.
